# Dominance of high-risk clones ST2 and ST571 and the diversity of resistance islands in clinical Acinetobacter baumannii isolates from Hanoi, Vietnam

**DOI:** 10.1099/mgen.0.001500

**Published:** 2025-09-17

**Authors:** Minh Ngoc Nghiem, Dung Phuong Bui, Van Thi Thu Ha, Hop Thi Tran, Diem Thi Nguyen, Thuy Thi Bich Vo

**Affiliations:** 1 Institute of Biology, Vietnam Academy of Science and Technology, 18 Hoang Quoc Viet, Nghia Do, Hanoi 100000, Vietnam; 2 VNU University of Science, 334 Nguyen Trai, Hanoi 100000, Vietnam; 3 Vietnam Medical Military University, 160 Phung Hung, Hanoi 100000, Vietnam

**Keywords:** *Acinetobacter baumannii*, antibiotic resistance genes, mobile genetic elements, multidrug resistance, virulence genes

## Abstract

Multidrug-resistant *Acinetobacter baumannii* poses a significant threat to hospital environments worldwide, including Vietnam. In this study, we conducted whole-genome sequencing on 30 clinical *A. baumannii* isolates from Hanoi to explore their genomic diversity, antibiotic resistance determinants, virulence factors and mobile genetic elements. Phylogenetic analyses, utilizing both SNP-based and multilocus sequence typing-based approaches, revealed that the isolates clustered into various sequence types (STs). Among these, ST2 and ST571 emerged as the dominant high-risk clones. The ST2 isolates exhibited a wide range of resistance genes, such as *bla*_OXA-23_, *mph(E*), *msr(E*) and *armA*. Additionally, they contained mobile genetic elements, including plasmids and AbaR-type resistance islands, which promote horizontal gene transfer. Virulence gene analysis showed the presence of several key determinants like *ompA*, *adeFGH* and *bfmRS* and quorum sensing regulators *abaI* and *abaR*, underscoring the strains’ potential for persistent colonization and infection. These findings highlight the marked genomic diversity and robust resistance profiles of Vietnamese *A. baumannii* isolates. The predominance of ST2 and ST571, corresponding to global clones GC2 and GC1, respectively, along with frequent co-occurrence of *bla*_OXA-23_ and *armA*, suggests region-specific features distinct from those reported in other parts of Southeast Asia. This underscores the need for improved surveillance and targeted infection control strategies.

**Impact Statement**

This study highlights the genetic diversity of *Acinetobacter baumannii* strains isolated from clinical samples in Hanoi, Vietnam. The findings indicate that these strains not only harbour multiple antibiotic resistance genes but also possess mobile genetic elements that facilitate the spread of resistance. Additionally, the presence of virulence factors suggests a potential survival advantage in the hospital environment. The analysis reveals a high prevalence of two specific sequence types, ST2 and ST571, underscoring the urgent need for enhanced surveillance and targeted infection control measures to effectively address the growing threat of multidrug-resistant *A. baumannii* in Vietnam.

## Data Summary

All sequencing data and supplementary materials have been deposited under BioProject accession number PRJNA1233432 and are available at the NCBI/SRA database (https://www.ncbi.nlm.nih.gov/sra/PRJNA1233432).

The whole-genome sequencing quality assessment of 30 *Acinetobacter baumannii* strains in this study is listed in Table S1 (available in the online Supplementary Material).

## Introduction

Multidrug-resistant *Acinetobacter baumannii* (*Ab*) is a pathogen frequently encountered in hospitals, posing a significant threat to public health, particularly in intensive care units. This bacterium is known to cause severe infections such as ventilator-associated pneumonia, septicaemia, meningitis and urinary tract infections, and it is responsible for cross-contamination in healthcare settings [1]. The spread of *Ab* has been reported globally, with occurrences noted in Europe, Asia-Pacific and North America [2]. In Vietnam, *Acinetobacter* species have been found in various environments, especially within hospitals, where they serve as reservoirs for antibiotic resistance genes (ARGs) [3].

Many strains of *Ab* exhibit resistance to multiple classes of antibiotics, including beta-lactams, aminoglycosides, tetracyclines and fluoroquinolones. These resistant strains typically carry ARGs on mobile genetic elements (MGEs) such as plasmids, transposons and integrons, which facilitate horizontal gene transfer (HGT) between bacteria [4]. Key resistance genes include bla_OXA-23_, encoding a class D carbapenemase; armA, a 16S rRNA methyltransferase conferring aminoglycoside resistance; and the msr(E)-mph(E) pair, mediating macrolide resistance through efflux and drug modification [4,5]. *oqxA* and *oqxB* encode multidrug efflux pumps, while *floR* confers phenicol resistance [6]. Plasmids, one of the key MGEs, are extrachromosomal DNA molecules capable of autonomous replication. Certain plasmids, such as AC716 identified in this study, can carry numerous resistance genes and facilitate the horizontal spread of multidrug resistance (MDR) across bacterial populations [7]. Resistance islands (RIs) are large chromosomal regions acquired through HGT, carrying clusters of resistance genes embedded within mobile elements such as transposons and integrons. Notable examples include AbaR-type islands (e.g. AbaR3 and AbaR4b), which are frequently found in global clone 2 strains and contribute to carbapenem resistance [8]. RIs, which contain transposons, integrons and ARGs, play a crucial role in the transmission of resistance traits, particularly among global clonal lineages. The majority of multidrug-resistant *Ab* strains belong to global clones 1 and 2. These global clones correspond to sequence types (STs) ST1 and ST2, respectively, defined using multilocus sequence typing (MLST) based on seven conserved housekeeping genes. ST2 is also referred to as global clone 2 (GC2) and ST1 as GC1 – both are epidemic lineages linked to MDR and hospital outbreaks [9].

Although several studies have characterized ARGs and virulence factors in *Ab*, research focusing on the genomic features of *Ab* strains in Vietnam remains limited. Previous epidemiological studies on *Ab* in Vietnam [10,11] have provided insights into the clinical aspects and prevalence of *Ab*, but a comprehensive genomic analysis is still lacking. The distribution of ARGs, virulence factors and MGEs in *Ab* strains remains poorly understood in Vietnam. These gaps highlight the urgent need for further research to investigate the genomic features of *Ab* strains isolated from hospitals in Vietnam.

This study hypothesizes that *Ab* strains isolated from clinical samples in Hanoi, Vietnam, possess a distinct genomic profile compared to globally distributed clones (e.g. GC1 and GC2) and previously reported Vietnamese isolates. Such differences may stem from regional antibiotic usage practices and hospital-specific selection pressures, which have been shown to influence the genomic structure and resistance profiles of *Ab* populations in various settings [2,9, 10].

A recent multicentre genomic study conducted across six hospitals in northern, central and southern Vietnam between 2017 and 2019 identified ST2 and ST571 as the most prevalent STs among carbapenem-resistant *Ab* isolates [12]. These findings suggest that high-risk clones had already been circulating across the country prior to our investigation.

Recent findings suggest that resistance and virulence genes may co-occur within the same genome or mobile element, potentially enhancing bacterial adaptability in hospital settings [9,13]. Exploring such convergence could improve our understanding of how multidrug-resistant pathogens evolve and persist. In this context, the present study focuses on *Ab* isolates collected between January and December 2022 from clinical samples at Military Hospital 103 and Military Central Hospital 108 in Hanoi, Vietnam. We hypothesize that these recent isolates may exhibit genomic features distinct from globally distributed clones (e.g. GC1/ST1 and GC2/ST2) and from earlier Vietnamese collections. Such differences could reflect regional antibiotic practices and hospital-specific selection pressures, which are known to influence the genomic structure and resistance profiles of *Ab*.

Therefore, our primary objective is to perform whole-genome sequencing (WGS) to investigate the resistance determinants, virulence factors and MGEs of these isolates. The results will be compared with global and regional data to identify any novel genomic features that may be unique to the Vietnamese context. This work aims to support more effective surveillance and infection control strategies.

## Methods

### Sample collection and bacterial identification

Thirty strains of *Ab* were isolated from clinical samples, including blood, urine, sputum, wound fluid, bronchial fluid and abdominal fluid. These samples were collected from patients at the Microbiology Departments of Military Hospital 103, Military Central Hospital 108, Vietnam, between January and December 2022. Medical microbiology specialists at these hospitals identified and confirmed the bacterial samples. The *Ab* strains were tested for antibiotic susceptibility using the Phoenix BD Line 58 automated bacterial identification and susceptibility testing system.

### DNA extraction, library preparation and WGS

Bacterial DNA was extracted according to the protocol described by Sambrook and Russell (2006) [14]. DNA libraries were prepared using the Illumina TruSeq DNA PCR-Free Kit following the manufacturer’s instructions. WGS was performed using the NexSeq 500 platform (Illumina). The quality of the raw sequencing data was assessed using Galaxy, an open-source bioinformatic platform (https://usegalaxy.org.au), developed by the University of Melbourne, Australia. The raw data were cleaned by removing low-quality reads (e.g. reads shorter than 200 bases or containing homopolymers) using the appropriate Galaxy tools. The high-quality data were then subjected to *de novo* genome assembly using SPAdes v3.15.2 available within the Galaxy platform. The quality of the assembled genomes was assessed based on total genome size, maximum contig length and N50 index using Galaxy’s QUAST integration. Genome annotation was performed using the Prokka tool within Galaxy.

### Bioinformatic analysis

MLST was performed using the PubMLST platform (2018) (https://pubmlst.org/organisms/acinetobacter-baumannii) based on the Pasteur method to identify the STs of the strains. The seven housekeeping genes used for MLST analysis were *cpn60*, *fusA*, *gltA*, *pyrG*, *recA*, *rplB* and *rpoB*. ARGs were annotated using ABRIcate v1.0.1 (https://github.com/tseemann/abricate) and the ResFinder database (https://cge.food.dtu.dk/services/ResFinder/). Virulence genes were identified from the VFDB database using Galaxy’s VFanalyzer tool. Plasmids in the assembled genomes were detected using MOB-suite v3.0.3 (https://github.com/phac-nml/mob-suite). The presence of recombinase integrons was identified using IntegronFinder v2.0 (https://github.com/gem-pasteur/Integron_Finder).

### Phylogenetic analysis and classification

The genomes of the 30 *Ab* strains were compared with reference genomes belonging to the same or closely related STs, including ACICU, BJAB0868, BJAB07104 and BJAB0715 using Roary v3.13.0 within Galaxy. Core genes were defined as those present in at least 99% of the samples and showing at least 95% similarity in aa sequence. Short-read sequencing data from the 30 samples were mapped to the reference genome of *Ab* ATCC17978, and SNPs were analysed using Snippy v4.6.0, also available through the Galaxy interface.

A phylogenetic tree was constructed using the maximum likelihood method with RaxML v8.2.12 integrated into Galaxy. This analysis employed the GTRGAMMA model and included 250 bootstrap replicates. The tree was built from three datasets: core genome, core SNP and whole-genome core sites, where ‘core sites’ were defined as positions that appeared in all samples, including both SNPs and invariant sites. The phylogenetic tree was visualized using iTOL, which is also accessible through Galaxy.

## Results

### WGS quality assessment

The whole genomes of 30 *Ab* strains were successfully sequenced with high-quality results. The recovery rate ranged from 97% to 99% compared with the pre-purified sequences. The guanine-cytosine content in the samples averaged 40 mol%, both before and after purification, indicating stable genome properties. The number of contigs varied from 76 to 1761, and the total genome length was between ~3.8 million bp and over 4.5 million bp. This aligns with the genome sizes of *Ab* strains documented in the NCBI database, where the N50 index varied from 4,823 to 164,357 bp (see Table S1).

**Fig. 1.**
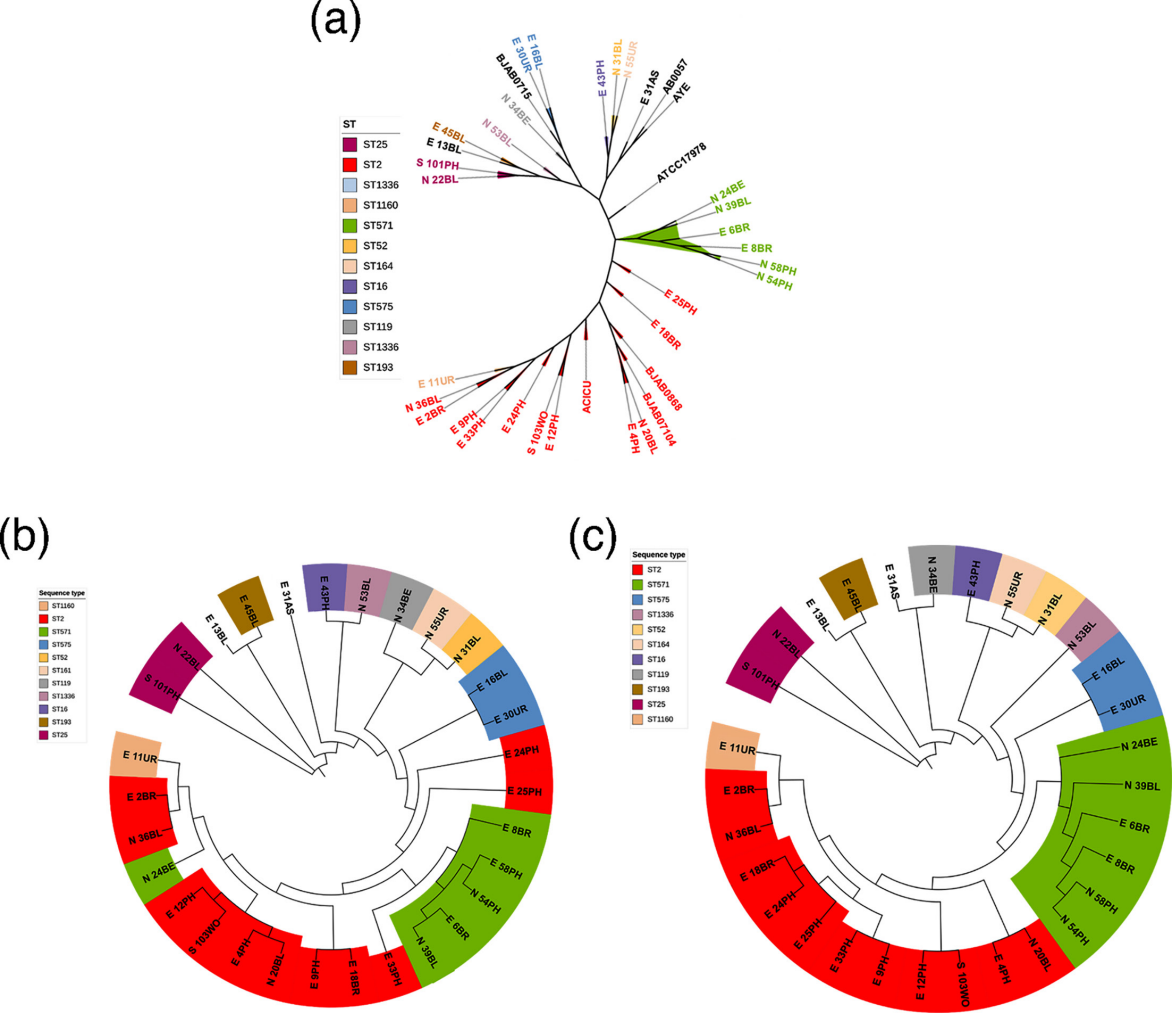
Classification tree based on the core genome (**a**), core SNP (**b**) and whole-genome core site (**c**).

The core genome-based phylogenetic tree (Fig. 1a) demonstrated a clear clustering of isolates into distinct STs. Notably, strains E13BL and E45BL were grouped within the ST193 clade. The ST571 clade included multiple strains, such as E6BR, E8BR and N24BE. The ST575 clade consisted of strains E30UR and E16BL, while the ST119 clade contained N34BE. Additionally, the ST25 clade was delineated by the clustering of S101PH and N22BL, and the ST2 group showed multiple sub-branches, indicating a notable level of genetic diversity within that lineage.

In contrast, the core SNP-based tree (Fig. 1b) and the whole-genome core site-based tree (Fig. 1c), which incorporates both SNPs and invariant sites, generally produced clustering patterns consistent with the core genome tree, although some variations were noted. A notable difference was observed in the placement of strain E31AS. In the core genome tree (Fig. 1a), E31AS was positioned in a distinct clade, whereas in the core SNP-based tree (Fig. 1b), it remained isolated. In the whole-genome core site-based tree (Fig. 1c), E31AS grouped with N34BE within ST119.

### MLST typing and ARG profiles

MLST analysis successfully identified STs for 28 out of 30 bacterial isolates, revealing 11 distinct STs. Among them, ST2 was the most prevalent, accounting for 11/30 strains (39.29%), followed by ST571 with 6/30 strains (21.43%). Notably, two strains, E13BL and E31AS, isolated from blood and sterile fluids, did not match any known STs in the database, indicating that they may represent potential novel STs.

**Fig. 2.**
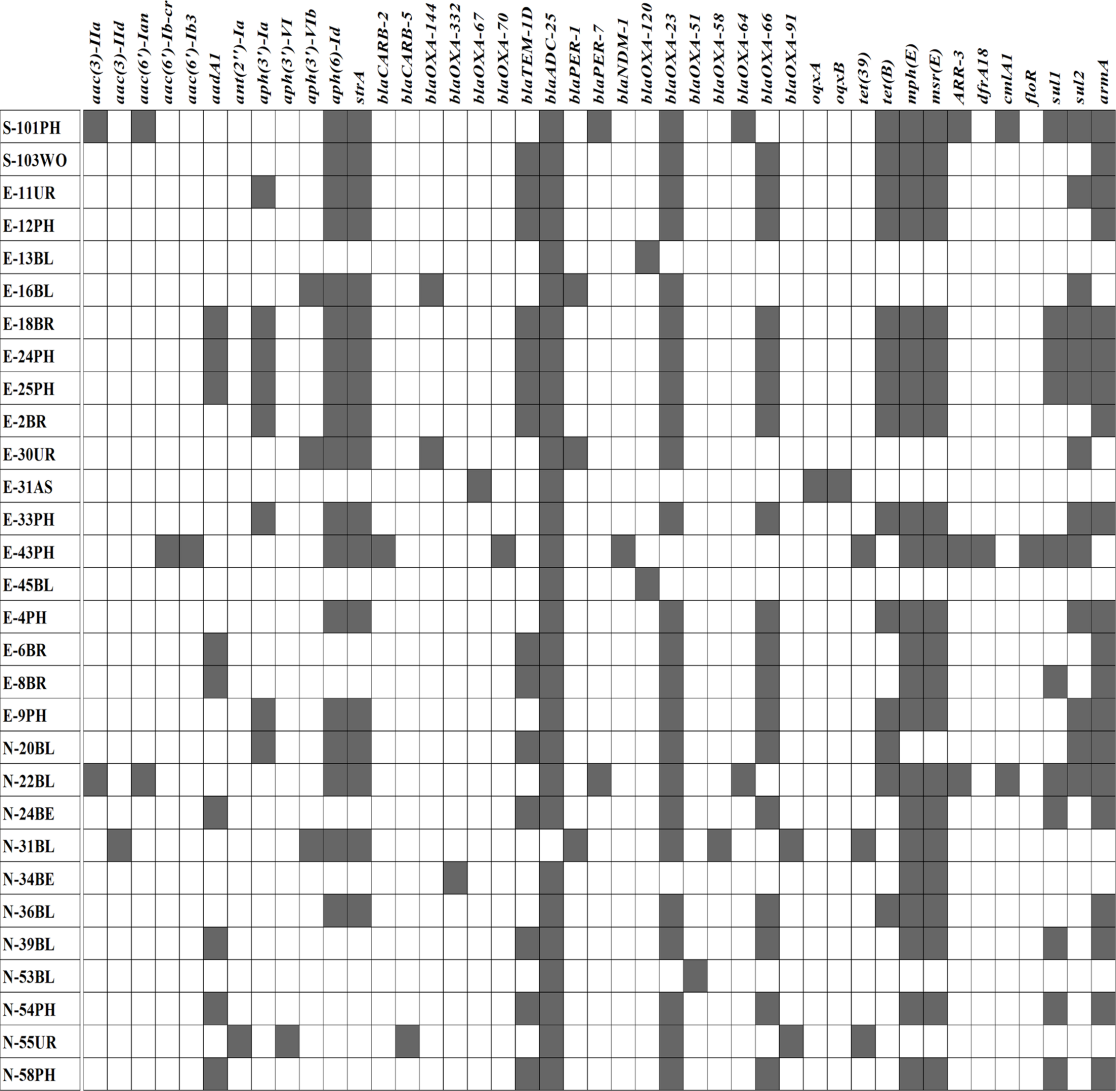
Distribution of ARGs in *Ab* isolates.

A total of 43 ARGs were detected across the isolates (Fig. 2). The most frequently found genes included *bla*_OXA-23_ (24/30), *mph(E*) and *msr(E*) (22/30) and *armA* (20/30), conferring resistance to beta-lactams, macrolides and aminoglycosides. Additionally, *strB*, *strA* and *bla*_OXA-66_ were present in 18/30 isolates. *bla*_ADC- 25_ was also found in 29/30 strains; however, as a chromosomally encoded housekeeping gene, its presence alone may not imply resistance. Although *bla*_OXA-51-like_ genes are typically intrinsic to *Ab*, they were only detected in one strain in this dataset. This may be due to annotation artefacts or assembly limitations, as short-read sequencing sometimes fails to capture complete chromosomal elements. Less common resistance genes, such as *aac(3)-IIa*, *aac(3)-IId*, *aac(6′)-Ian aac(6′)-Ib-cr*, *bla*_CARB-2_, *bla*_PER-1_, *bla*_NDM-1_ and *bla*_OXA-58_, were detected sporadically among different isolates, suggesting variations in resistance mechanisms. Tetracycline resistance was linked to the presence of *tet(B*) and *tet(39),* while sulphonamide resistance was associated with *sul1* (11 strains) and *sul2* (13 strains). Additionally, *ARR-3* was identified in isolates resistant to rifamycins, while *oqxA* and *oqxB* were linked to MDR, and *clmA1* and *floR* conferred resistance to phenicol antibiotics.

Most *Ab* isolates exhibited MDR, possessing genes that conferred resistance to at least three antibiotic classes. The E43PH strain (ST16) and S101PH and N22BL strains (ST25) were found to carry up to 16 resistance genes, indicating broad-spectrum resistance. Notably, the E43PH strain was the only one harbouring *aac(6′)-Ib-cr*, *aac(6′)-Ib3*, *bla*_CARB-2_, *bla*_OXA-70_, *bla*_NDM-1_ and *floR*. The E18BR, E24PH and E25PH strains (ST2) possessed 14 resistance genes, underscoring the high resistance potential within this lineage.

**Fig. 3.**
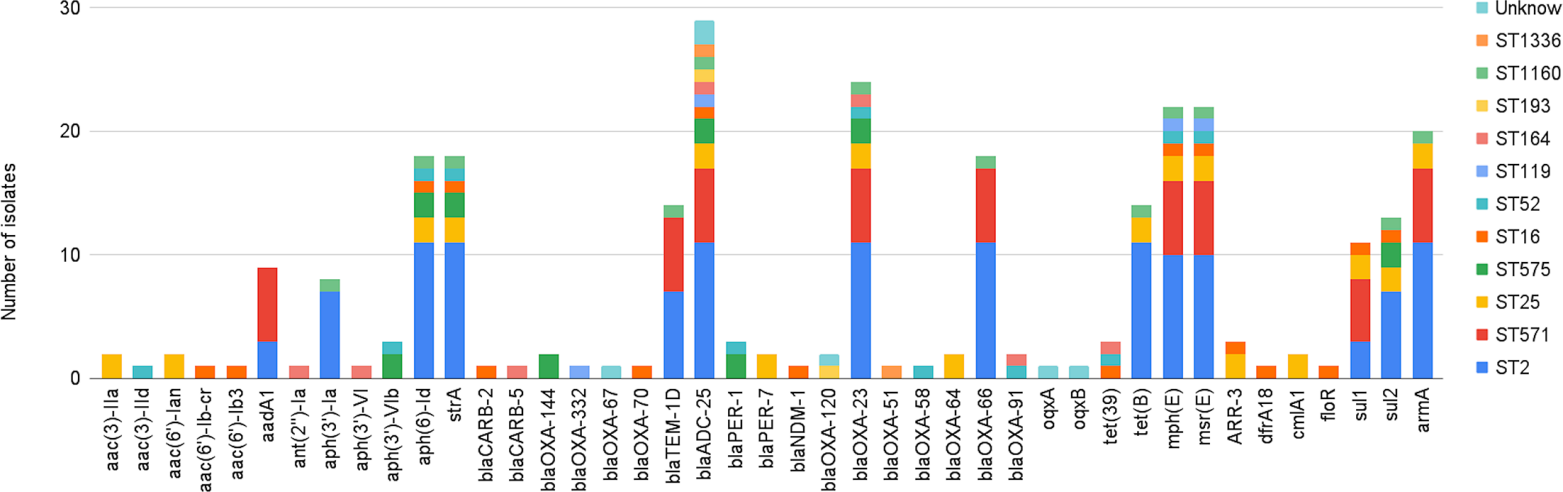
Distribution of ARGs according to STs.

These results indicate that the SNP-based approach yields a higher-resolution phylogenetic tree, enabling a more detailed differentiation among closely related bacterial isolates compared with the MLST-based methods. Concerning the ST distribution of ARGs in the 30 strains, we observed that all ST2 strains contained the *strB*, *strA*, *bla*_ADC-25_, *bla*_OXA-23_, *bla*_OXA-66_, *tet(B*), *mph(E*), *msr(E*) and *armA* genes (Fig. 3). ST2 strains carry a varying number of ARGs, ranging from 9 to 14 genes. Two isolates, S-101PH and N-22BL, belonging to ST25, exhibited identical ARGs. A total of five out of six strains from ST571 had identical ARGs, except for the E-6BR isolate, which lacked the *sul1* gene. Two unidentified STs of E-13BL and E-31AS strains had significantly fewer resistance genes than the others; however, the E-31AS strain was the only one that carried the *oqxA* and *oqxB* genes.

### Virulence genes

**Fig. 4.**
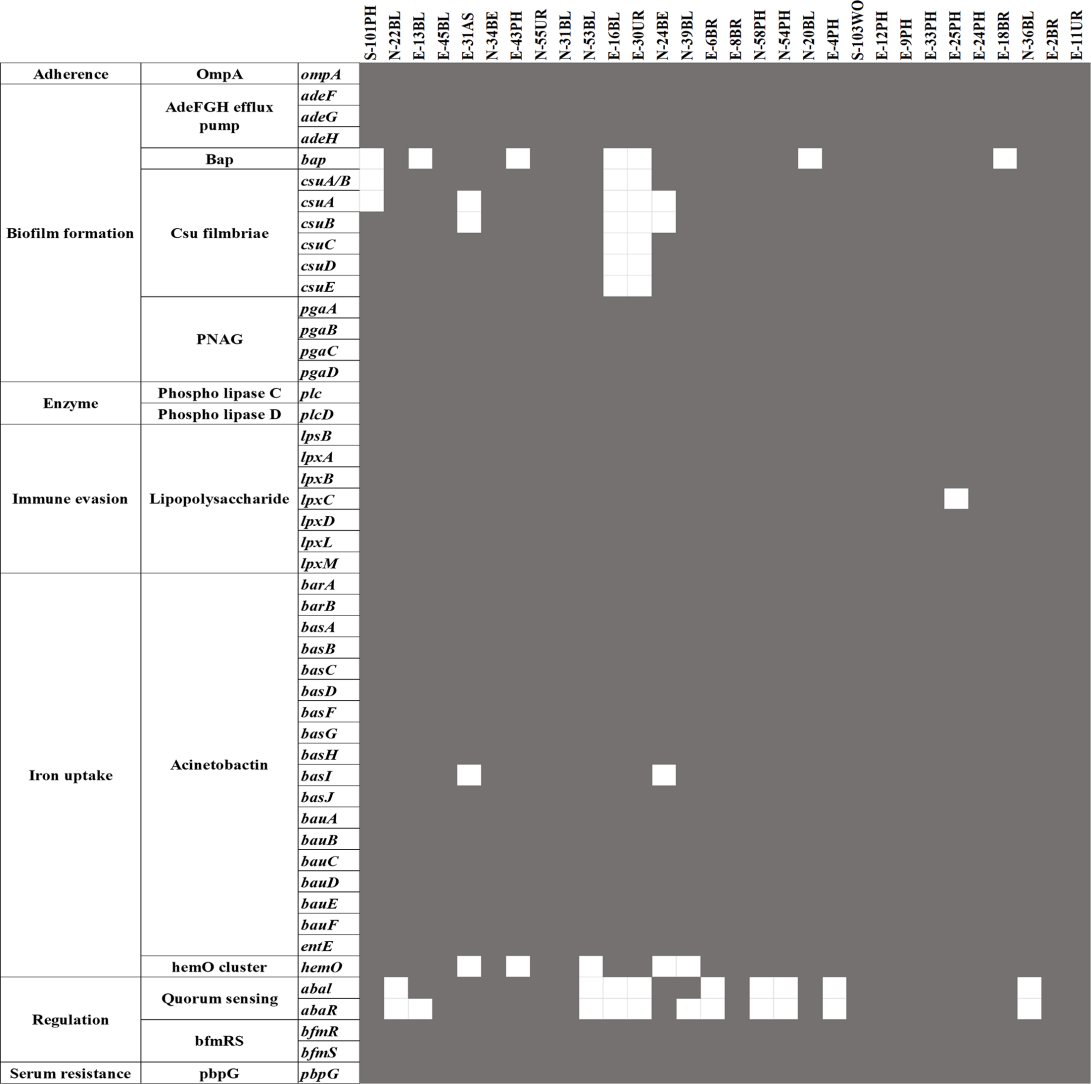
Distribution of virulence genes in *Ab* isolates.

A total of 48 virulence genes were identified across the strains, categorized into adherence, biofilm formation, immune evasion, iron uptake, regulation and serum resistance (Fig. 4). All 12 strains harboured mutations in all 48 genes. The genes *ompA*, *adeFGH* efflux pump, PNAG, phospholipase D/C, lipopolysaccharide, *bfmRS* and pbpG were found in every strain. Additionally, 21 out of 30 strains carried both *abaI* and *abaR* genes, which are associated with quorum sensing regulation. However, the *csu*A/B/C/D/E genes that encode Csu pili and the *bap* gene were absent in strains E-16BL and E-30UR.

### MGE related to ARGs: plasmids and RIs

**Fig. 5.**
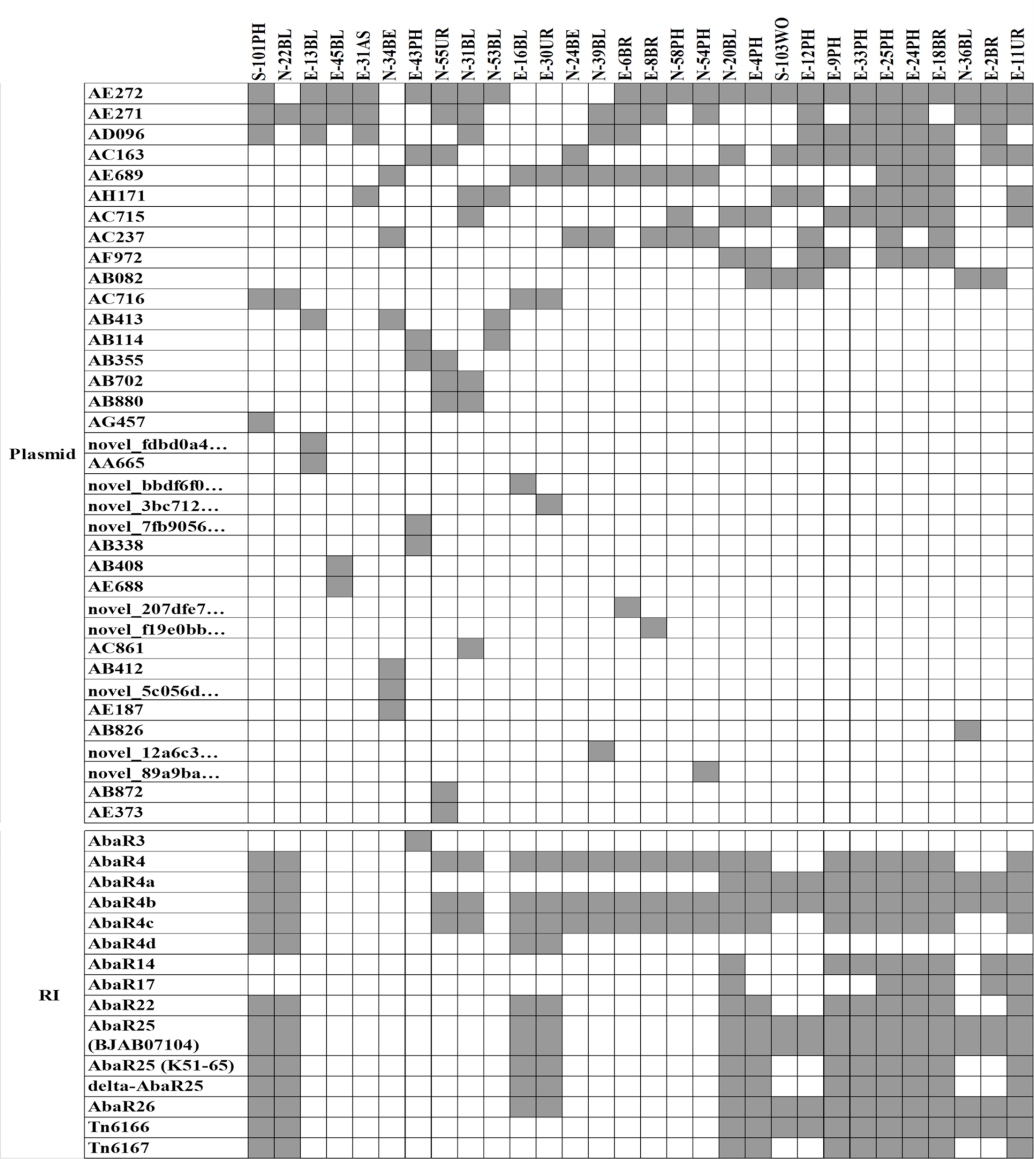
Distribution of plasmids and RIs in *Ab* isolates.

A total of 36 plasmids were identified across 30 strains, with 10 of these plasmids detected in at least 5 strains (Fig. 5). Among them, 19 plasmids were found to carry ARGs, predominantly *bla*_OXA_ genes. The AE272 plasmid was the most frequently observed, present in 24 strains, but interestingly, it did not contain any ARGs. In contrast, the AC716 plasmid, which was found in four strains, harboured 12 ARGs. The resistance genes encoded in these plasmids primarily conferred resistance to beta-lactams and aminoglycosides, although resistance genes for sulphonamide, tetracycline and phenicol were also identified (Table 1). WGS revealed the presence of 15 RIs. The strains N-20BL, E-25PH, E-24PH, E-18BR and E-11UR had the highest number of RIs, with each carrying 13. Strains S-101PH, N-22BL (ST25) and E-9PH, E-33PH (ST2) harboured 12 RIs each. In contrast, strains E-13BL and E-31AS (unassigned STs), E-45BL (ST193) and N-34BE (ST119) lacked any detectable RIs. Additionally, the transposons Tn6166 and Tn6167 were found in 14 strains, primarily belonging to ST2 (*n*=10), ST571 (*n*=3) and ST16 (*n*=1). An RI structurally similar to AbaR4, lacking a full Tn6022 core and carrying IS26 insertions, was found in 24 isolates (mainly ST2), while a structure resembling AbaR3 was identified in the ST16 strain E-43PH.

**Table 1.** Details of predicted plasmids from MOB-suite

Plasmid	ARG (in all isolates containing plasmids)
AE272	
AE271	*blaOXA*-91_1, *blaOXA*-120_1, *blaOXA*-67_1, *blaOXA*-66_1
AC163	*blaOXA*-23_1
AD096	*mph(E)_*1, *msr(E)_*1, *armA*_1
AE689	*blaOXA*-23_1
AC715	*blaOXA*-23_1, *aph(3')-*VIb_1, *sul2*_2, *tet(B)_*1, *aph(6)-*Id_1, *aph(3'')-*Ib_2, *sul1*_5, *ant(3'')-*Ia_1
AH171	
AC237	*sul1*_5, *armA*_1, *msr(E)_*1, *mph(E)_*1, *blaTEM*-1D_1, *ant(3'')-*Ia_1, *aph(3')-*Ia_7
AF972	
AB082	*blaOXA*-23_1
AC716	*mph(E)_*1, *msr(E)_*1, *armA*_1, *aac(6')-*Ian_1, *aac(3)-*IIa_1, *aph(3''*)-Ib_2, *aph(6)-*Id_1, *ARR*-3_4, *cmlA1*_1, *blaPER*-7_1, *tet(B*)_1, *sul1*_5, *sul2*_2
AB413	
AB702	*ant(2''*)-Ia_13
AB114	*floR*_2, *sul2*_2, *msr(E*)_1, *mph(E*)_1
AB355	*tet(39*)_1
AB880	
AG457	*aac(3*)-IIa_1
AE373	*aph(3')-*VI_1
AC861	*aph(6*)-Id_1, *mph(E)_*1, *msr(E)_*1, *aac(3)-*IId_1
novel_12a6c3e2aa896865d52105c3a512fa08	*blaOXA*-23_1
novel_207dfe76ce26e19a7b48fd531546ec09	*blaOXA*-23_1
novel_3bc7128461026ecd275d380a6d6c4e2e	*blaOXA*-23_1
novel_bbdf6f0e1a3b520c977560c2d8e4694e	*blaOXA*-23_1
novel_f19e0bb1c1d87ddd7be8c2c69cf27e78	*blaOXA*-23_1
AA665	
AB338	
AB408	
AB412	
AB826	
AB872	
AE187	
AE688	
novel_5c056d5ca175dc725a432bbade889fd8	
novel_7fb9056a1ed08de2b69dda1c3b6330f6	

### Co-occurrence and co-localization of resistance and virulence genes

To explore the relationship between ARGs and virulence factors, we examined their distribution across the 30 *Ab* isolates. A notable pattern was observed between the carbapenem resistance gene *bla*_OXA-23_ (present in 24/30 isolates) and the quorum sensing regulator *abaR* (21/30), which frequently co-occurred within the same strains. Similarly, the aminoglycoside resistance gene *armA* (20/30) often appeared in isolates carrying *bfmRS*, a key transcriptional regulator found universally in this dataset. In contrast, widely distributed genes such as *bla*_ADC-25_ (29/30) and *ompA* did not show any specific co-distribution pattern.

In terms of physical linkage, plasmid and RI analysis revealed that the AC716 plasmid (found in four isolates) carried both *bla*_OXA-23_ and *abaR*, suggesting potential co-localization of resistance and regulatory functions. The AbaR4b RI, present in 24 isolates, frequently carried *bla*_OXA-23_ along with *abaI* (18/30), pointing to a mechanism for joint dissemination of resistance and quorum sensing traits. In contrast, the AE272 plasmid, though prevalent (24/30), lacked both resistance and virulence genes, highlighting the functional diversity of mobile elements within the *Ab* genome.

## Discussion

The results from WGS revealed marked genetic diversity among the 30 *Ab* strains studied, with ST distribution showing a predominance of ST2 and ST571. ST2 is widely recognized as a high-risk clonal lineage associated with MDR and global dissemination [9]. In our study, ST2 accounted for 39.29% of the strains, which aligns with previous research highlighting its clinical importance in nosocomial infections [15]. Similarly, ST571 made up 21.43% of the strains and has been increasingly reported in hospital outbreaks, particularly in cases involving carbapenem-resistant *Ab* [10]. The high prevalence of these STs suggests that they are well adapted to withstand antimicrobial pressures and have a strong ability to persist in healthcare environments.

A key observation in this study was the correlation between ST and antibiotic resistance profiles. All ST2 isolates contained a core set of resistance genes, including *bla*_OXA-23_, *bla*_OXA-66_, *strB*, *strA*, *tet(B*), *mph(E*), *msr(E*) and *armA*. These genes confer resistance to beta-lactams, aminoglycosides, tetracyclines and macrolides. The high prevalence of *bla*_OXA-23_, a significant carbapenemase gene, is particularly concerning, as it is a major contributor to carbapenem resistance in *Ab* globally [16]. The co-occurrence of *mph(E*) and *msr(E*) in multiple strains suggests that macrolide resistance may be more widespread than previously recognized. A study analysing azithromycin-resistant *Escherichia coli* and *Salmonella* isolates from various animal sources found that the *msr(E)-mph(E*) gene pair was present in all animal groups examined, indicating a broad distribution of these resistance determinants [5]. Interestingly, the two unclassified STs (E-13BL and E-31AS) showed notably fewer resistance genes, with E-31AS uniquely harbouring *oqxA* and *oqxB* genes, which contribute to MDR through efflux mechanisms [6].

In addition to individual resistance genes, the presence of RIs and plasmids highlights the role of HGT in the development of antimicrobial resistance. Our study identified 36 plasmids, 19 of which carry resistance genes, including several variants of *bla*_OXA_. The AC716 plasmid was detected in 4 strains and contained 12 resistance genes, emphasizing the role of plasmid-mediated dissemination in *Ab* resistance evolution [7]. Additionally, the detection of AbaR4b in 24 isolates supports earlier research, showing that AbaR-type RIs are characteristic of MDR *Ab* [8]. While our findings confirm the importance of these genetic elements in acquiring resistance, more functional studies are needed to understand their specific contributions to observable resistance.

Virulence analysis showed that a wide range of virulence genes are present across all strains, including *ompA*, *adeFGH*, *bfmRS* and *pbpG*. These genes are essential for processes such as adherence, biofilm formation and immune evasion [17]. Importantly, quorum sensing regulators *abaI* and *abaR* were detected in 21 out of 30 isolates, suggesting a potential role in regulating virulence and biofilm development. The absence of csuA/B/C/D/E and bap in the E-16BL and E-30UR strains suggests possible changes in their ability to form biofilms, which may affect colonization and persistence [18].

The co-occurrence of resistance genes (*bla*_OXA-23_ and *armA*) and virulence regulators (*abaR*, *abaI* and *bfmRS*) in ST2 and ST571 strains suggests a convergent adaptation strategy favouring both antimicrobial survival and enhanced colonization. This convergence has been observed in global high-risk clones and is increasingly recognized as a factor driving clinical persistence of *Ab* [9].

The identification of *bla*_OXA-23_ and *abaR* on the same plasmid (AC716), and of *bla*_OXA-23_ with *abaI* on the AbaR4b RI, suggests the potential for co-transfer of resistance and virulence genes via MGEs. The regulatory systems *abaI/abaR* and *bfmRS* are known to control biofilm formation, quorum sensing and virulence expression, which may increase fitness under hospital conditions [13,19].

Interestingly, MGEs such as AE272, although widely distributed, did not carry either resistance or virulence genes, highlighting the heterogeneity of MGEs in *Ab*. While short-read sequencing limits the confirmation of physical linkage, the frequent co-occurrence of resistance and virulence determinants in MGE-positive strains suggests a need for long-read genome analysis. Understanding how MGEs shape the spread of pathogenic traits is essential for infection control and antibiotic stewardship strategies.

The predominance of ST2 and ST571 in our isolates aligns with global reports, where ST2 is widely recognized as a dominant multidrug-resistant clone in Asia and Europe [8,9, 15]. ST571 has also been increasingly reported in Southeast Asia [10]. Based on MLST analysis, ST571 is a double-locus variant of ST2, differing at only two housekeeping loci. This close relationship suggests that both STs may share core resistance and virulence features, while exhibiting minor genomic variations. Although both ST2 and ST571 were dominant in our dataset, phylogenetic analysis revealed that they form distinct clades with different RI structures and gene content. ST2 is a well-characterized international clone, whereas ST571 appears to be more geographically restricted and less extensively studied.

Notably, the co-localization of *bla*_OXA-23_ and *abaR* on plasmid AC716 and the widespread presence of AbaR4b suggest possible local adaptations of global clones. While similar MGEs have been observed globally, recent studies also highlight the acquisition of *bla*_NDM_ and novel RIs in ST2 strains from other regions, indicating ongoing evolution under antibiotic pressure [7,8].

## Conclusion

This study demonstrates that clinical *Ab* isolates from Hanoi display significant genetic diversity, with ST2 and ST571 being the predominant high-risk clones. These strains carry multiple ARGs and MGEs that facilitate the spread of resistance, along with key virulence factors that enhance their survival in hospital settings. Our findings underscore the urgent need for improved surveillance and targeted infection control measures to address the threat posed by multidrug-resistant *Ab* in Vietnam.

## Supplementary material

10.1099/mgen.0.001500 Uncited Table S1.

## Abbreviations

*Ab*: *Acinetobacter baumannii*
ARG: antibiotic resistance gene
HGT: horizontal gene transfer
MDR: multidrug resistance
MGE: mobile genetic element
MLST: multilocus sequence typing
RI: resistance island
ST: sequence type
WGS: whole-genome sequencing
